# The Impact of Exercise Intensity and Sex on Endogenous Ghrelin Levels and Appetite in Healthy Humans

**DOI:** 10.1210/jendso/bvae165

**Published:** 2024-10-24

**Authors:** Kara C Anderson, Tana Mardian, Benjamin Stephenson, Emily E Grammer, Macy E Stahl, Nathan R Weeldreyer, Zhenqi Liu, Kaitlin M Love, Sibylle Kranz, Jason D Allen, Arthur Weltman

**Affiliations:** Department of Kinesiology, School of Education and Human Development, University of Virginia, Charlottesville, VA 22904, USA; Department of Medicine, University of Virginia Health System, Charlottesville, VA 22908, USA; Department of Kinesiology, School of Education and Human Development, University of Virginia, Charlottesville, VA 22904, USA; Department of Kinesiology, School of Education and Human Development, University of Virginia, Charlottesville, VA 22904, USA; Department of Kinesiology, School of Education and Human Development, University of Virginia, Charlottesville, VA 22904, USA; Department of Kinesiology, School of Education and Human Development, University of Virginia, Charlottesville, VA 22904, USA; Department of Kinesiology, School of Education and Human Development, University of Virginia, Charlottesville, VA 22904, USA; Department of Medicine, University of Virginia Health System, Charlottesville, VA 22908, USA; Department of Medicine, University of Virginia Health System, Charlottesville, VA 22908, USA; Department of Kinesiology, School of Education and Human Development, University of Virginia, Charlottesville, VA 22904, USA; Department of Kinesiology, School of Education and Human Development, University of Virginia, Charlottesville, VA 22904, USA; Department of Medicine, University of Virginia Health System, Charlottesville, VA 22908, USA; Department of Kinesiology, School of Education and Human Development, University of Virginia, Charlottesville, VA 22904, USA; Department of Medicine, University of Virginia Health System, Charlottesville, VA 22908, USA

**Keywords:** acute exercise, gut hormones, appetite regulation, lactate kinetics, sex differences

## Abstract

**Context:**

Ghrelin circulates in acylated (AG) and deacylated (DAG) forms, which are known to affect appetite. Although acute exercise has been shown to modulate ghrelin levels, data on the impact of exercise intensity on AG and DAG levels and their effects on appetite are sparse and primarily limited to males.

**Objective:**

To investigate the effect of exercise intensity and sex on ghrelin levels and appetite in untrained humans.

**Methods:**

Eight males (age: 43.1 ± 10.9 years; body mass index [BMI]: 22.2 ± 1.7 kg/m^2^; peak oxygen consumption [VO_2peak_]: 36.3 ± 6.4 mL/kg/min) and 6 females (age: 32.2 ± 11.1 years; BMI: 22.7 ± 1.0 kg/m^2^; VO_2peak_: 29.2 ± 4.0 mL/kg/min) completed a maximal graded cycle ergometer lactate threshold (LT)/VO_2peak_ test. These data were used to determine the exercise intensity on 3 subsequent randomized control or calorically matched cycle exercise bouts: (1) CON, no exercise; (2) MOD, the power output at LT; (3) HIGH, the power output associated with 75% of the difference between LT and VO_2peak_. Perception of appetite was analyzed using visual analog scales.

**Results:**

Females had higher levels of total ghrelin (TG) (*P* = .03) and DAG (*P* = .01) at baseline than males. Both groups exhibited reduced DAG levels in HIGH compared with MOD and CON (*P* < .0001-.004); however, only females had significantly reduced AG in HIGH (*P* < .0001). Hunger scores were higher in MOD than in CON (*P* < .01).

**Conclusion:**

High-intensity may be superior to moderate-intensity exercise for reducing ghrelin levels and modifying hunger, and sex may impact this response.

Ghrelin was discovered in 1999 by Kojima and Kangawa as an endogenous ligand to the growth hormone secretagogue receptor 1a (GHSR1a) [1]. Although early work focused on ghrelin's ability to stimulate growth hormone release, ghrelin has since been shown to have wide-ranging biological effects in areas including energy balance, appetite, glucose homeostasis, immune function, sleep, and memory [2].

Ghrelin exists in 2 forms, acylated (AG) and deacylated (DAG), with the majority circulating as DAG [3] (∼78% of total ghrelin [TG]). Although less abundant, AG (∼22% of TG [3]) is better characterized as it is known to bind to GHSR1a and is catalyzed by ghrelin O-acyltransferase (GOAT) [2]. DAG was initially thought to be inactive but is now known to bind to an unidentified receptor with independent biological effects. The ability to differentiate between AG and DAG and their physiological signaling is important to determine if they act in an independent, antagonistic, or synergistic manner within the body [2]. The effects of ghrelin on appetite highlight the divergent effects of the 2 forms. While AG has stimulated appetite in human and animal models [4-6], DAG has been shown to either have no effect or to suppress appetite [7, 8].

This particular effect of ghrelin is of interest in exercise studies, as acute exercise of appropriate intensity can suppress appetite in healthy populations and in populations with obesity [9, 10]. However, literature on the link between exercise, appetite, and ghrelin levels is poorly defined. This may be due to the complex interplay of several variables including exercise dose, isoform measured, the impact of sex, and different feeding states that could confound data interpretation. Additionally, previous studies have typically included only healthy, young adult men, thus limiting real-world applicability [11, 12].

Identifying the ideal exercise dose for appetite suppression may have clinical utility as a weight loss intervention. A study by Vanderheyden et al used sodium bicarbonate supplementation to investigate lactate's role in exercise-induced ghrelin suppression. They found that higher blood lactate levels (via sodium bicarbonate) suppressed AG levels and decreased perception of appetite, suggesting that lactate is involved in the suppression of ghrelin [13]. Therefore, exercise intensity may be a key modulator of this relationship, as elevated levels of blood lactate seen with high intensities may suppress AG and appetite after exercise. A recent meta-analysis published by our group determined that exercise suppresses all isoforms of ghrelin, and that exercise intensity moderates that relationship; however, most studies included in the meta-analysis utilized a moderate-intensity exercise bout and sampled only healthy males [14].

Evidence also suggests that ghrelin concentrations differ between biological sex, where females may have higher levels of AG and DAG than males [15, 16]. Limited data have also found estrogen may be at least partly responsible [17, 18]. Results regarding the effect of exercise are equivocal; most studies show no differences in ghrelin levels between sexes in response to exercise; however, protocols only employed 1 exercise intensity [19, 20]. To our knowledge, this is the first study to investigate sex differences in each ghrelin isoform with differing exercise intensities.

Therefore, the purpose of this study was to investigate the effects of exercise intensity on appetite and ghrelin levels and assess sex differences. We hypothesized that high-intensity exercise will lead to the greatest alterations in ghrelin levels (ie, decrease in AG levels) and suppression of appetite, and that females will have higher levels of all ghrelin isoforms than males.

## Materials and Methods

### Participants

Individuals between the ages of 18 and 55 years were recruited for this study. They were selected for screening if they were untrained, nonsmoking, and weight stable (<3 kg over 3 months) and had a body mass index (BMI) of 18.5 to 24.9 kg/m^2^. Criteria for exclusion included history of type 2 diabetes, pregnancy/fertility treatments, disorders of the endocrine and gastrointestinal system, and/or any medications/treatments that affected the ability to safely exercise or measure hormones. Individuals arrived at the University of Virginia Clinical Research Unit (CRU) between 7 and 9 Am after an overnight fast for all visits. Subjects were asked to refrain from strenuous exercise and alcohol consumption for 24 hours, and tobacco products and caffeine use for 12 hours prior to each CRU admission. The study was conducted in accordance with the Declaration of Helsinki, the protocol was approved by the University of Virginia Institutional Review Board (IRB-HSR # 200241), and all subjects provided written informed consent.

### Screening Period

Subjects’ percent body fat (BF%) was assessed via dual energy x-ray absorptiometry (Hologic Horizon). Peak oxygen consumption (VO_2peak_) and lactate threshold (LT) were determined via an incremental test on a cycle ergometer (Lode Model 960900). Subjects began at an initial power output of 50 W and power output was increased 25 W every 3 minutes until volitional fatigue. Indirect calorimetry using standard open circuit spirometry (Vyaire Medical, Viasys, Yorba Linda, CA) was used to measure oxygen consumption and carbon dioxide production (also to provide minute by minute kcal to equate caloric expenditure between the 2 exercise bouts). Blood was sampled via an indwelling catheter placed in an antecubital vein at the end of each stage and assayed for lactate (YSI Instruments 2900, Yellow Springs, OH, USA). The LT was determined as the power output just before the curvilinear increase in blood lactate (LAC); the VO_2_ at this power output was chosen as VO_2_ LT. The highest 1 minute segment VO_2_ attained was chosen as VO_2peak_.

### Testing Period

**Figure 1.**
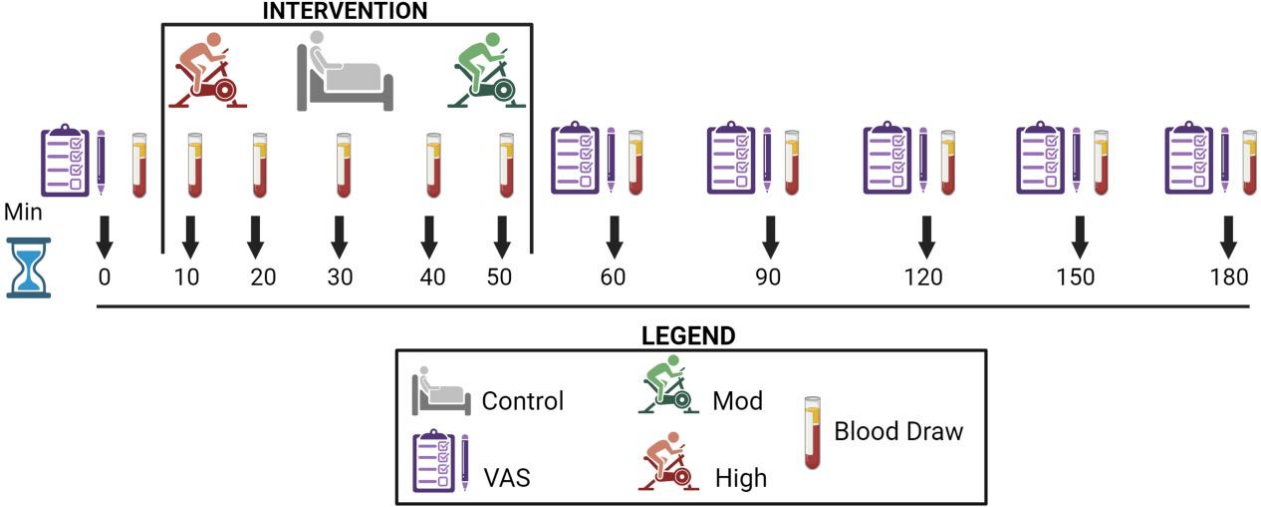
Outline of testing visit measurements.

Figure 1 presents an outline for testing visits. The testing period consisted of 3 randomized visits: control (CON, no exercise), moderate-intensity exercise (MOD, power output at LT), and high-intensity exercise (HIGH, power output associated with 75% of the difference between LT and peak). Females were tested during the early follicular phase of the menstrual cycle, which was verbally confirmed. There was a minimum of 72 hours between exercise sessions for males, and females completed their visits monthly to standardize to the menstrual cycle. Participants recorded their diet for the day before the first testing visit and were asked to replicate the same diet for each testing visit.

Caloric expenditure was matched within each subject for both exercise conditions. At each visit, subjects were observed for 3 hours. An indwelling catheter was inserted into the antecubital vein and blood was sampled at baseline, every 10 minutes for the first hour, and then every 30 minutes for the remaining 2 hours to measure TG, AG, DAG, and lactate. Appetite ratings, hunger (HUN), satisfaction (SAT), fullness (FULL), and desire to eat (EAT), were measured via a 100 mm visual analogue scale (VAS) at baseline and every 30 minutes thereafter. The VAS was composed of a series of identical lines with anchors on the end of each line (ie, “I have never been more hungry”/“I am not hungry at all”). Subjects made a mark along the line, corresponding to their perception of each domain to quantify their feelings. A score for each rating (hunger, satisfaction, fullness, and desire to eat) was calculated by measuring the distance from the left end of the line to the mark [21]. A total appetite score (APP) at each timepoint was calculated with the formula:


$$\begin{array}{rl} \mathrm{Appetite}\;= & \left(\mathrm{Desire}\;\mathrm{to}\;\mathrm{eat}+\mathrm{Hunger}+(100-\mathrm{Fullness}\right)\\ & +(100-\mathrm{Satisfaction}))/4 \end{array}$$

### Biochemical Analyses

Blood for LAC was immediately analyzed (YSI Instruments 2900). Blood to measure TG, AG, and DAG was collected in 3 mL EDTA vacutainers containing 0.06 mL of protease inhibitor AEBSF and was centrifuged for 10 minutes at 3000 rpm at 4 °C. A 100-mL bolus of hydrochloric acid 1N was added to the plasma aliquots immediately after centrifugation. Plasma ghrelin was stored at −80 °C for later analysis. Ghrelin was analyzed using Bertin Pharma ELISA kits by the University of Virginia Center for Research in Reproduction, Ligand Assay and Analysis Core. The intraassay variability for AG (RRID:AB_2936966) was 4.4% with a minimum detection limit of 3.0 pg/mL; for DAG (RRID:AB_2819343) the variability was 2.9% with a minimum detection limit of 2.0 pg/mL. All samples were run in duplicate. Net incremental area under the curve (AUC) was calculated for TG, AG, DAG, and each appetite VAS measure, and total AUC was calculated for LAC from each testing visit via the trapezoidal method.

### Statistics

Based on previous literature [15], assuming a power of 80% for an ANOVA with a significance of α = .05, and an effect size of .5, an adequate sample size of n = 8 per group was determined a priori to assess group differences between acute exercise and ghrelin levels. Power calculations were made with G*Power version 3.1. Data were analyzed via R (Version 2023.06.1+524). Baseline comparisons were evaluated using independent sample t-tests, and normality was assessed using Q-Q plots and the Shapiro–Wilk tests.

Several linear mixed models were used to examine the ghrelin response to exercise. For each timepoint (TG_TIMEPOINT_, AG_TIMEPOINT_, DAG_TIMEPOINT,_ LAC_TIMEPOINT,_ HUN_TIMEPOINT_, FULL_TIMEPOINT_, SAT_TIMEPOINT_, EAT_TIMEPOINT_, APP_TIMEPOINT_) the following models were examined: subject as a random factor and sex (male or female), condition, and time were fixed factors. For AUC (TG_AUC_, AG_AUC,_ DAG_AUC,_ LAC_AUC,_ HUN_AUC_, FULL_AUC_, SAT_AUC_, EAT_AUC_, APP_AUC_) data: subject was a random factor and sex and condition were fixed factors. Satterwhite's approximation was utilized to determine significance. F-tests of nested models were used to determine differences in fixed effects. Estimated marginal means were utilized to estimate the means that were adjusted for the factors in each model. Associations were determined using Spearman's rank correlations if data were not normal and/or the relationship between variables of interest was not linear, and Pearson product–moment correlations were utilized in normal and linearly related data. Significance was set a priori as *P* ≤ .05. Data from linear mixed models are reported as mean ± SE; data in tables are reported as mean ± SD.

## Results

Eight males (age: 43.1 ± 10.9 years; BMI: 22.2 ± 1.7 kg/m^2^; VO_2peak_: 36.3 ± 6.4 mL/kg/min) and 6 females (age: 32.2 ± 11.1 years; BMI: 22.7 ± 1.0 kg/m^2^; VO_2peak_: 29.2 ± 4.0 mL/kg/min) completed the study. Sample characteristics are shown in Table 1. Females had significantly higher BF% (*P* = .0004), baseline DAG (*P* = .01), and TG levels (*P* = .03), while males had a higher VO_2peak_ (*P* = .02). Regarding the exercise sessions, males had a higher energy expenditure across conditions and a higher power output during HIGH than females (both, *P* = .02). Due to significant differences in baseline ghrelin levels and hunger scores across conditions within females, the values for each VAS component are presented as delta scores from baseline.

**Table 1.** Sample demographics

	Males		Females	*P* value
N	8		6	
Age (years)	43.1 ± 10.9		32.2 ± 11.1	.09
BMI (kg/m^2^)	22.2 ± 1.7		22.7 ± 1.0	.51
Total BF (%)	22.0 ± 4.27		34.4 ± 4.8	.0004
VO_2peak_ (mL/kg/min)	36.3 ± 6.4		29.2 ± 4.0	.02
VO_2peak_ (L/min)	2.4 ± 0.4		1.8 ± 0.5	.02
Baseline AG (pg/mL)	94.1 ± 52.92		125.57 ± 66.30	.16
Baseline DAG (pg/mL)	98.53 ± 40.61		143.46 ± 58.53	.01
Baseline TG (pg/mL)	196.0 ± 83.71		274.23 ± 133.76	.03
Peak lactate (mM/L)	0.43 ± 0.14		0.46 ± 0.17	.52
Hunger	42.60 ± 22.80		41.94 ± 21.24	.92
Satisfaction	28.46 ± 16.93		28.06 ± 16.36	.93
Fullness	26.77 ± 15.62		24.41 ± 19.51	.68
Desire to eat	58.29 ± 24.50		61.71 ± 18.88	.61
Total appetite	60.77 ± 16.06		62.79 ± 14.21	.67
**Exercise sessions**	Mod	High	Mod	High
Duration (min)*^a^*	55:19 ± 0.3	32:06 ± 0.2	53:29 ± 0.1	35:44 ± 0.2
Energy expenditure (kcal)	314.2 ± 27.9	315.5 ± 30.0*^b^*	274.2 ± 27.5	266.8 ± 24.6*^b^*
Average HR (bpm)*^a^*	116.95 ± 7.0	164.9 ± 13.08.07	130.6 ± 20.6	170.7 ± 20.7
Average power output (watts)*^a^*	78.6 ± 10.8	147.9 ± 30.1*^b^*	65.8 ± 11.7	106.1 ± 16.55
%VO_2peak_*^a^*	68.9 ± 6.1	89.1 ± 3.45 *^b^*	72.8 ± 5.75	94.3 ± 1.4

Abbreviations: AG, acylated ghrelin; DAG, deacylated ghrelin; TG, total ghrelin; VO_2peak_, peak oxygen consumption.

^*a*^Difference between MOD and HIGH, *P* < .0001.

^*b*^Difference within the same condition between males and females, *P* = .02.

### Total Ghrelin

**Figure 2.**
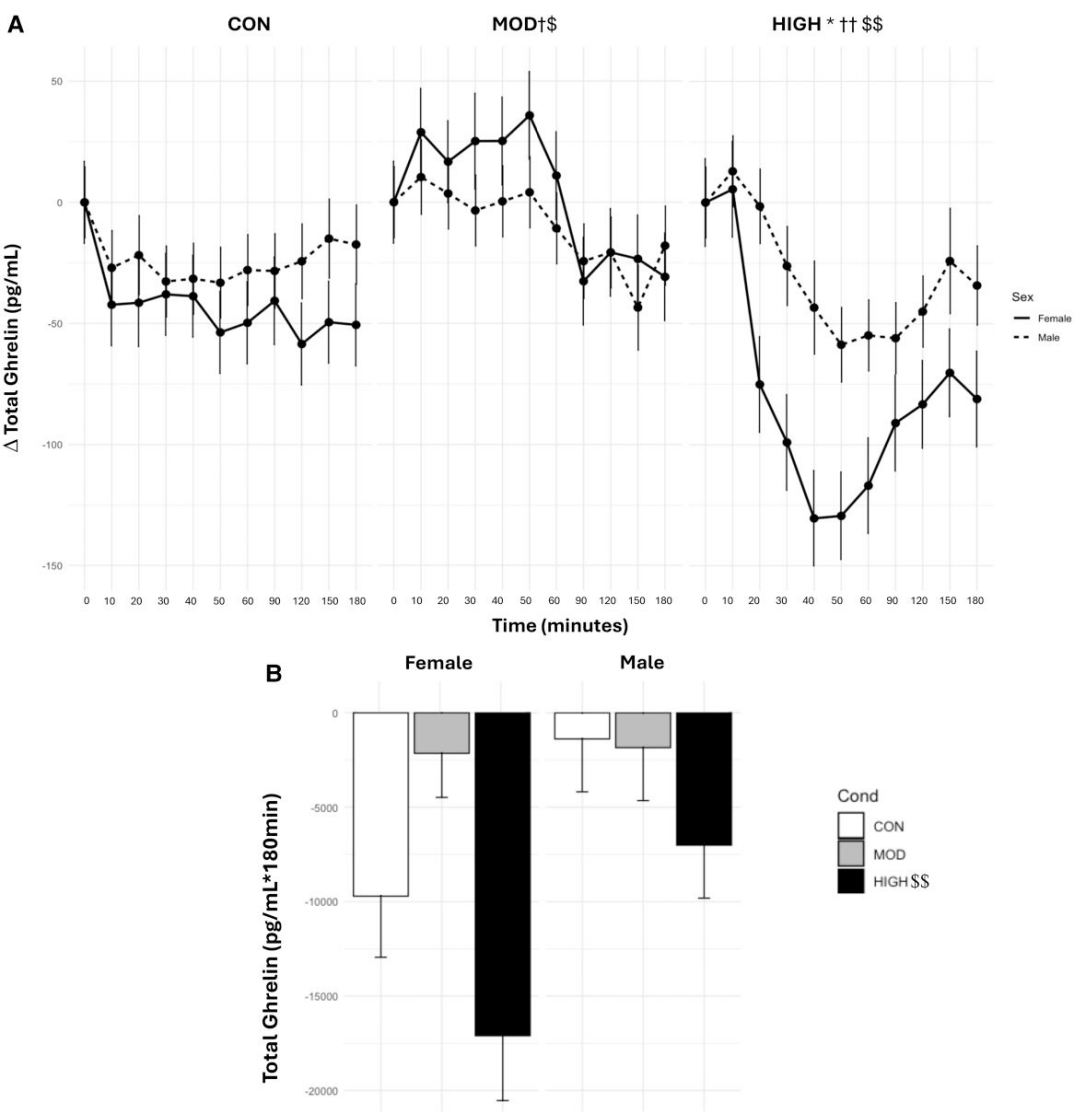
Effect of condition on (A) TG_TIMEPOINT_ and (B) TG_AUC_. Data are mean ± SE. The statistical analysis revealed a main effect of condition (*P* < .0001), where levels of TG_TIMEPOINT_ were lower in HIGH than in MOD and CON. TG_TIMEPOINT_ levels were also significantly higher in MOD than in CON (*P* < .0001). There was a main effect of time (*P* < .0001), where TG_TIMEPOINT_ levels were lower during 40 to 180 minutes compared with baseline. There were significant interaction effects between condition and time and condition and sex (both *P* < .0001). HIGH had reduced TG levels at 40 to 120 minutes compared with baseline. Males had higher levels of TG_TIMEPOINT_ in MOD compared to HIGH. Females had significantly lower levels of TG in HIGH than in CON and MOD. Females also had lower TG in CON than MOD and lower TG levels in HIGH compared with males in HIGH (*P* = .03). For TG_AUC_, there was a main effect for condition (*P* = .003), where levels of TG_AUC_ were significantly lower in HIGH than in MOD and CON. *Significantly different from MOD in males, *P* = .006. †Significantly different from CON in females, *P* < .0001. ††Significantly different from CON and MOD in females, *P* < .0001. $Significantly different from CON, *P* < .0001. $$Significantly different from MOD and CON, *P* ≤ .01.

There was a main effect of condition (*P* < .0001), where levels of TG_TIMEPOINT_ were lower in HIGH (Δ −54.6 ± 7.24) than in MOD (Δ−2.91 ± 7.09) and CON (Δ−28.72 ± 7.04, both *P* < .0001). TG_TIMEPOINT_ levels were also significantly higher in MOD than in CON (*P* < .0001). There was a main effect of time (*P* < .0001), where TG _TIMEPOINT_ levels were lower during 40 to 180 minutes compared with baseline (*P* = <.0001-.005) (Fig. 2).

There were significant interaction effects between condition and time and condition and sex (both *P* < .0001). HIGH had reduced TG levels at 40 to 120 minutes compared with baseline (*P* < .0001-.05). Males had higher levels of TG_TIMEPOINT_ in MOD (Δ−9.07 ± 9.24) than in HIGH (Δ−29.9 ± 9.42, *P* = .006). Females had significantly lower levels of TG in HIGH (Δ−79.3 ± 11.01) than in CON (Δ−38.4 ± 10.6) and MOD (Δ3.24 ± 10.75, both *P* < .0001). Females also had lower TG in CON than in MOD (*P* < .0001) and lower TG levels in HIGH than males in HIGH (*P* = .03).

For TG_AUC_, there was a main effect for condition (*P* = .003), where levels of TG_AUC_ were significantly lower in HIGH (−12 055.0 ± 2177.0 pg/mL × 180 minutes) than in MOD (−1996.0 ± 2140.0 pg/mL × 180 minutes, *P* = .0002), and CON (−5945.0 ± 2140.0 pg/mL × 180 minutes, *P* = .01). The interaction between condition and sex did not reach statistical significance (*P* = .06), where TG_AUC_ in HIGH (−17 100.0 ± 3437.0) was lower than MOD (−2146.0 ± 3176.0, *P* = .002) in females.

### Acylated Ghrelin

**Figure 3.**
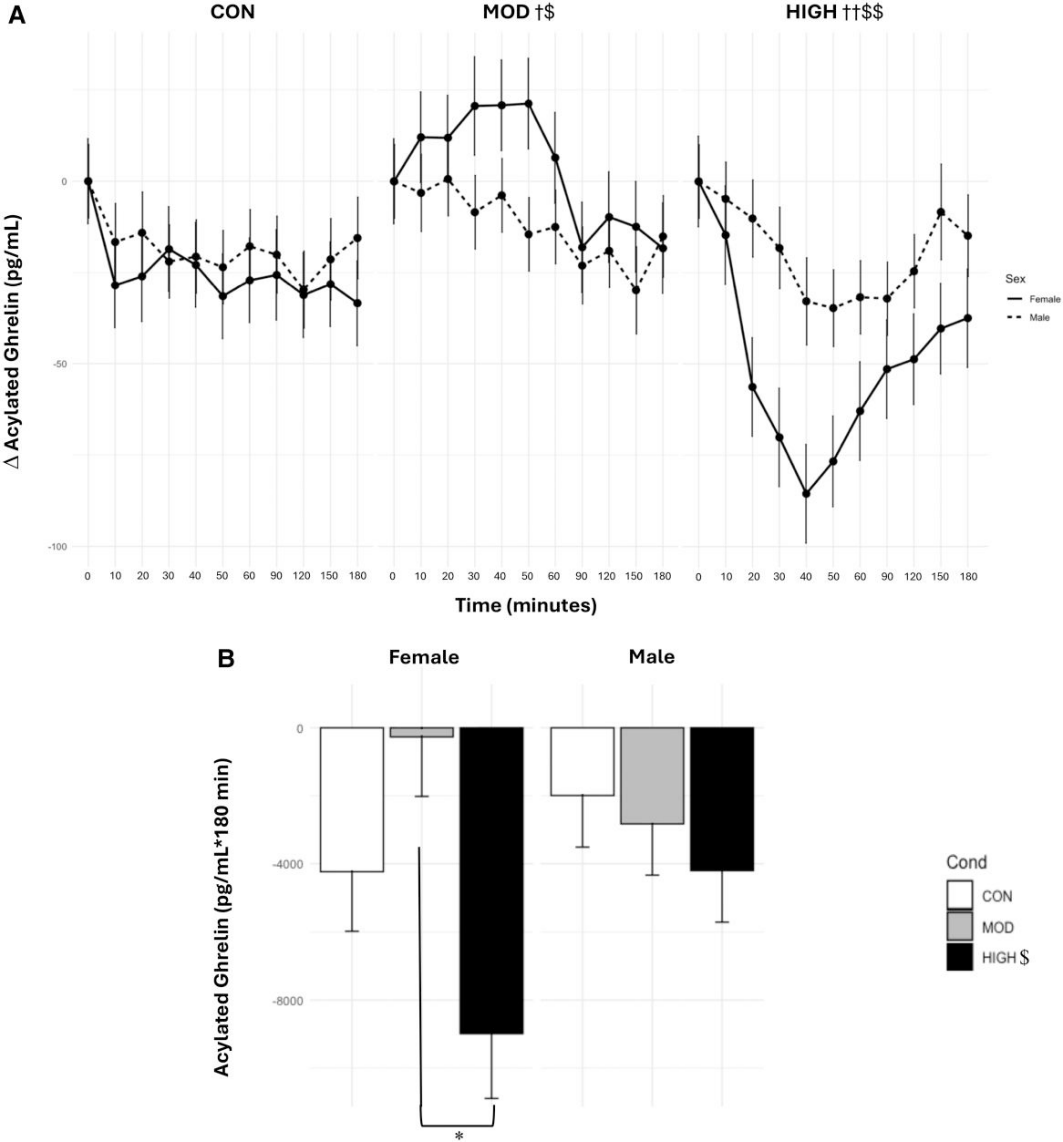
Effect of condition on (A) AG_TIMEPOINT_ and (B) AG_AUC_. Data are mean ± SE. The statistical analysis revealed there was a main effect of condition (*P* < .0001), where levels of AG_TIMEPOINT_ were lower in HIGH than in MOD and CON. AG_TIMEPOINT_ levels were also significantly higher in MOD than in CON. There was a main effect of time (*P* < .0001), where AG_TIMEPOINT_ levels were lower during 40 to 180 minutes compared with baseline. There were significant interaction effects between condition and time and condition and sex (both *P* < .0001). HIGH had significantly decreased AG levels at 30 to 90 minutes compared with baseline. Females had lower AG_TIMEPOINT_ levels in HIGH than in MOD and CON. AG levels in MOD were also significantly higher than in CON. There was no significant difference across any condition in males. There was a significant main effect of condition for AG_AUC_ (*P* < .005), where levels of AG_AUC_ were significantly lower in HIGH than in MOD and CON. There was also an interaction effect between condition and sex (*P* < .0001), where female AG_AUC_ in HIGH was significantly lower than MOD. *Significantly different within females, *P* < .0001. †Significantly different from CON in females, *P* < .006. ††Significantly different from CON and MOD in females, *P* < .0001. $Significantly different from CON, *P* < .0006. $$Significantly different from MOD and CON, *P* ≤ .005.

There was a main effect of condition (*P* < .0001), where levels of AG_TIMEPOINT_ were lower in HIGH (Δ−34.3 ± 4.6) than in MOD (Δ−4.21 ± 4.5) and CON (Δ−15.1 ± 4.5, both *P* < .0001). AG_TIMEPOINT_ levels were also significantly higher in MOD than in CON (*P* < .0006). There was a main effect of time (*P* < .0001), where AG_TIMEPOINT_ levels were lower during 40 to 180 minutes compared with baseline (*P* = <.0001-.01) (Fig. 3).

There were significant interaction effects between condition and time and condition and sex (both *P* < .0001). HIGH had significantly decreased AG levels at 30 to 90 minutes compared with baseline (*P* = .0001-.04). Females had lower AG_TIMEPOINT_ levels in HIGH (Δ−49.7 ± 7.1) than in MOD (Δ3.12 ± 6.7) and CON (Δ−22.01 ± 6.8, both *P* < .0001). AG levels in MOD were also significantly higher than CON (*P* < .0001). There was no significant difference across any condition in males (*P* = .61-.99).

There was a significant main effect of condition for AG_AUC_ (*P* < .005), where levels of AG_AUC_ were significantly lower in HIGH (−6593.0 ± 1215.0 pg/mL × 180 minutes) than in MOD (−1543.0 ± 1153.0 pg/mL × 180 minutes, *P* = .004) and CON (−3112.0 ± 1155.0 pg/mL × 180 minutes). There was also an interaction effect between condition and sex (*P* < .0001), where female AG_AUC_ in HIGH (−8987.0 ± 1901.0) was significantly lower than in MOD (−267.0 ± 1747.0, *P* = .006).

### Deacylated Ghrelin

**Figure 4.**
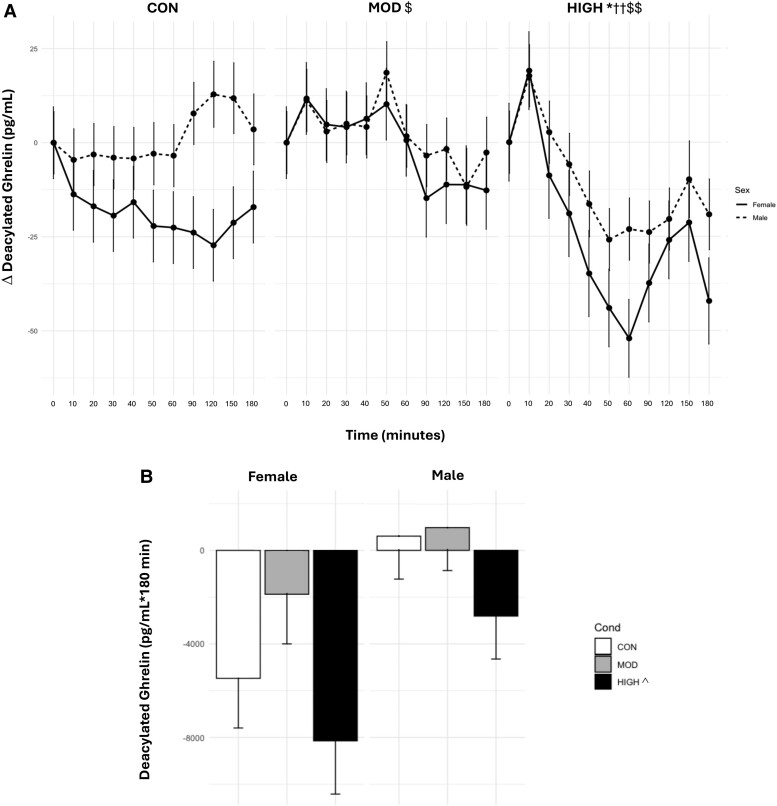
Effect of condition on (A) DAG_TIMEPOINT_ and (B) DAG_AUC_. Data are mean ± SE. The statistical analysis revealed there was a main effect of condition (*P* < .0001), where levels of DAG_TIMEPOINT_ were lower in HIGH than in MOD and CON. DAG_TIMEPOINT_ levels were also significantly higher in MOD than in CON. There was a main effect of time (*P* < .01), where DAG levels were lower during 60 minutes compared with baseline. A main effect for sex indicated females had lower DAG_TIMEPOINT_ levels regardless of condition than males. There was a significant interaction effect between condition and time (*P* < .0001) and condition and sex (*P* = .007). DAG was significantly lower at 50 and 60 minutes compared with baseline in HIGH. Males had lower levels of DAG_TIMEPOINT_ in HIGH than in MOD and CON. Females had lower DAG_TIMEPOINT_ levels in HIGH than in MOD and CON. CON in females was lower than CON in males. For DAG_AUC,_ there was a significant main effect of condition (*P* = .01), where levels of DAG_AUC_ were significantly lower in HIGH than in MOD. *Significantly different from CON and MOD in males, *P* ≤ .004. ††Significantly different from CON and MOD in females, *P* ≤ .0002. $Significantly different from CON, *P* = .001. $$Significantly different from MOD and CON, *P* ≤ .002. ^Significantly different from MOD, *P* = .008.

There was a main effect of condition (*P* < .0001), where levels of DAG_TIMEPOINT_ were lower in HIGH (Δ−17.7 ± 3.08) than in MOD (Δ .56 ± 2.99, *P* < .0001) and CON (Δ−8.46 ± 2.96, *P* = .002). DAG_TIMEPOINT_ levels were also significantly higher in MOD than in CON (*P* = .001). There was a main effect of time (*P* < .01), where DAG levels were lower during 60 minutes compared with baseline (*P* = .03). A main effect for sex indicated females had lower DAG_TIMEPOINT_ levels regardless of condition (Δ−14.5± 4.05) than males (Δ−1.58 ± 3.49, *P* = .03) (Fig. 4).

There was a significant interaction effect between condition and time (*P* < .0001) and condition and sex (*P* = .007). DAG was significantly lower at 50 (*P* = .02) and 60 minutes (*P* = .006) compared with baseline in HIGH. Males had lower levels of DAG_TIMEPOINT_ in HIGH (Δ−18.2 ± 4.45, *P* = .001) than in MOD (Δ2.20 ± 3.91, *P* = .001) and CON (1.26 ± 3.91, *P* = .004). Females had lower DAG_TIMEPOINT_ levels in HIGH (Δ−24.1 ± 4.76) than in MOD (−1.07 ± 4.5, *P* < .0001) and CON (−18.8 ± 4.45, both *P* = .0002). CON in females was lower than CON in males (*P* = .04).

For DAG_AUC,_ there was a significant main effect of condition (*P* = .01), where levels of DAG_AUC_ were significantly lower in HIGH (−5478.0 ± 1462.0 pg/mL × 180 minutes) than in MOD (−453.0 ± 1403.0 pg/mL × 180 minutes, *P* = .008). There was a nonsignificant main effect of sex (*P* = .056), where females had higher levels of DAG than males (−5166.0 ± 1715.0 vs −410.0 ± 1466.0 pg/mL × 180 minutes).

### Lactate

**Figure 5.**
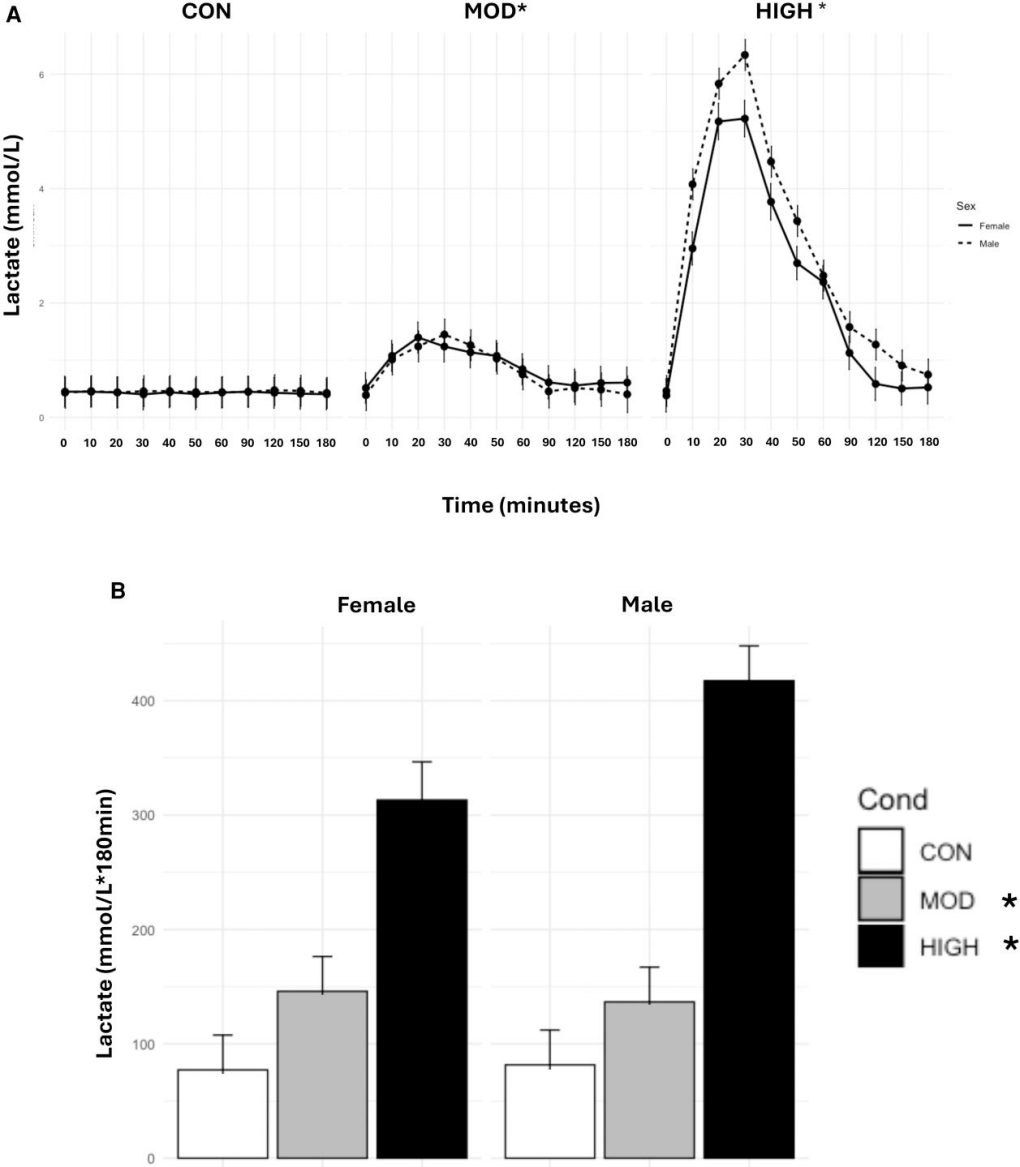
Effect of condition on (A) LAC_TIMEPOINT_ and (B) LAC_AUC_. Data are mean ± SE. The statistical analysis revealed there was a main effect of condition (*P* < .0001), where levels of LAC_TIMEPOINT_ were elevated in HIGH compared with MOD and CON. Levels were higher in MOD than in CON (*P* < .0001). There was a main effect of time (*P* < .0001), where LAC levels were higher during 10 to 90 minutes compared with baseline. There was significant condition by time and condition by sex interactions (both *P* < .0001). LAC_TIMEPOINT_ was elevated from 10 to 90 minutes compared with baseline in HIGH. LAC_TIMEPOINT_ was higher from 20 to 60 minutes compared with baseline during MOD. Females and males had higher levels of LAC during HIGH compared with MOD and CON and MOD compared with CON. For LAC_AUC,_ there was a main effect of condition (*P* < .001), where HIGH had elevated levels compared with MOD and CON. *Significantly different from the other 2 conditions *P* < .0001.

There was a main effect of condition (*P* < .0001), where levels of LAC_TIMEPOINT_ were elevated in HIGH (2.59 ± .1 mM/L) compared with MOD (0.85 ± .1 mM/L) and CON (0.44 ± .1 mM/L, both *P* < .0001). Levels were also higher in MOD than in CON (*P* < .0001). There was a main effect of time (*P* < .0001), where LAC levels were higher during 10 to 90 minutes compared with baseline (all *P* < .0001) (Fig. 5).

There was also significant condition by time and condition by sex interactions (both *P* < .0001). LAC_TIMEPOINT_ was elevated from 10 to 90 minutes compared with baseline in HIGH (all *P* < .0001). LAC_TIMEPOINT_ was higher from 20 to 60 minutes compared with baseline during MOD (*P* < .0001-.004). Females and males had higher levels of LAC during HIGH compared with MOD and CON and MOD compared with CON (all *P* < .0001).

For LAC_AUC,_ there was a main effect of condition (*P* < .001), where HIGH had elevated levels compared to MOD and CON (both *P* < .0001). The comparison of MOD and CON was not statistically significant (*P* = .07). There was no main effect of sex (*P* > .05).

### Hunger

**Figure 6.**
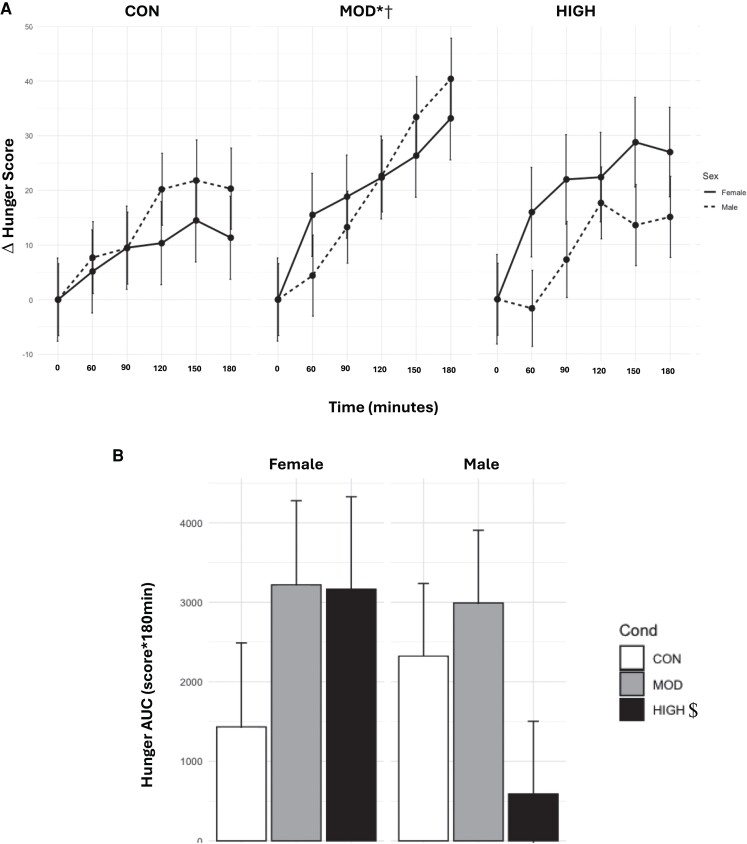
Effect of condition on (A) HUN_TIMEPOINT_ and (B) HUN_AUC_. Data are mean ± SE. The statistical analysis revealed there was a main effect of condition (*P* < .0001), where HUN_TIMEPOINT_ was higher in MOD than in CON. There was a main effect of time (*P* < .0001), where scores were lower during 90 to 180 minutes compared with baseline. There was a significant interaction between condition and sex (*P* = .01). Males had significantly higher HUN_TIMEPOINT_ in MOD than in HIGH. Females had significantly higher HUN_TIMEPOINT_ in MOD than in CON. *Significantly different from HIGH in males *P* = .05. †Significantly different from CON in females, *P* = .05.

There was a main effect of condition (*P* < .0001), where HUN_TIMEPOINT_ was higher in MOD (Δ19.2 ± 3.12) than in CON (Δ10.9 ± 3.11, *P* < .01). There were no differences between HUN_TIMEPOINT_ in HIGH (Δ14.1 ± 3.20) and CON (*P* = .45) or MOD (*P* = .14). There was a main effect of time (*P* < .0001), where scores were lower during 90 to 180 minutes compared with baseline (*P* = <.0001-.005) (Fig. 6).

There was a significant interaction between condition and sex (*P* = .01). Males had significantly higher HUN_TIMEPOINT_ in MOD (Δ19.01 ± 4.17) than in HIGH (Δ8.64 ± 4.15, *P* = .05), with no differences between CON (Δ13.24 ± 4.12) and HIGH (*P* = .77) or CON and MOD (*P* = .57). Females had significantly higher HUN_TIMEPOINT_ in MOD (Δ19.36 ± 4.65) than in CON (Δ8.47 ± 4.65, *P* = .05). The higher HUN_TIMEPOINT_ in HIGH (Δ19.57 ± 4.87) than in CON did not reach statistical significance (*P* = .07). There were no significant main or interaction effects for HUN_AUC_ (*P* = .19-.51).

### Satisfaction

There was significant main effect for time (*P* = .003); SAT_TIMEPOINT_ was significantly lower at 180 minutes compared with baseline (*P* = .02). Although SAT_TIMEPOINT_ in MOD (Δ−4.67 ± 2.07) was lower than CON (Δ−0.72 ± 2.06), it did not reach statistical significance (*P* = .08). There were no significant main or interaction effects for SAT_AUC_ (*P* = .44-.79). There was no main effect for sex in any model (*P* > .05). There were no significant main or interaction effects for SAT_AUC_ (*P* = .37-.90).

### Fullness

There were significant main effects for time (*P* < .001). The main effect for condition, where FULL_TIMEPOINT_ was lower in HIGH (Δ−4.23 ± 2.4) than in CON (Δ0.15 ± 2.34), did not reach the level of statistical significance (*P* = .06). FULL_TIMEPOINT_ was significantly lower at 180 minutes compared with baseline (*P* = .05). There were no significant main or interaction effects for FULL_AUC_ (*P* = .38-.72). There was no main effect for sex in any model (*P* > .05). There were no significant main or interaction effects for FULL_AUC_ (*P* = .44-.90).

### Desire to Eat

There were significant main effects for condition (*P* = .001), time (*P* = .002), but not sex (*P* > .05). EAT_TIMEPOINT_ was significantly higher in MOD (Δ9.76 ± 2.33) than in CON (Δ3.28 ± 2.32, *P* = .003). EAT_TIMEPOINT_ was also significantly lower at 150 (*P* = .007) and 180 minutes (*P* = .008) compared with baseline. There were no significant main or interaction effects for EAT_AUC_ (*P* = .32-.64).

### Total Appetite

**Figure 7.**
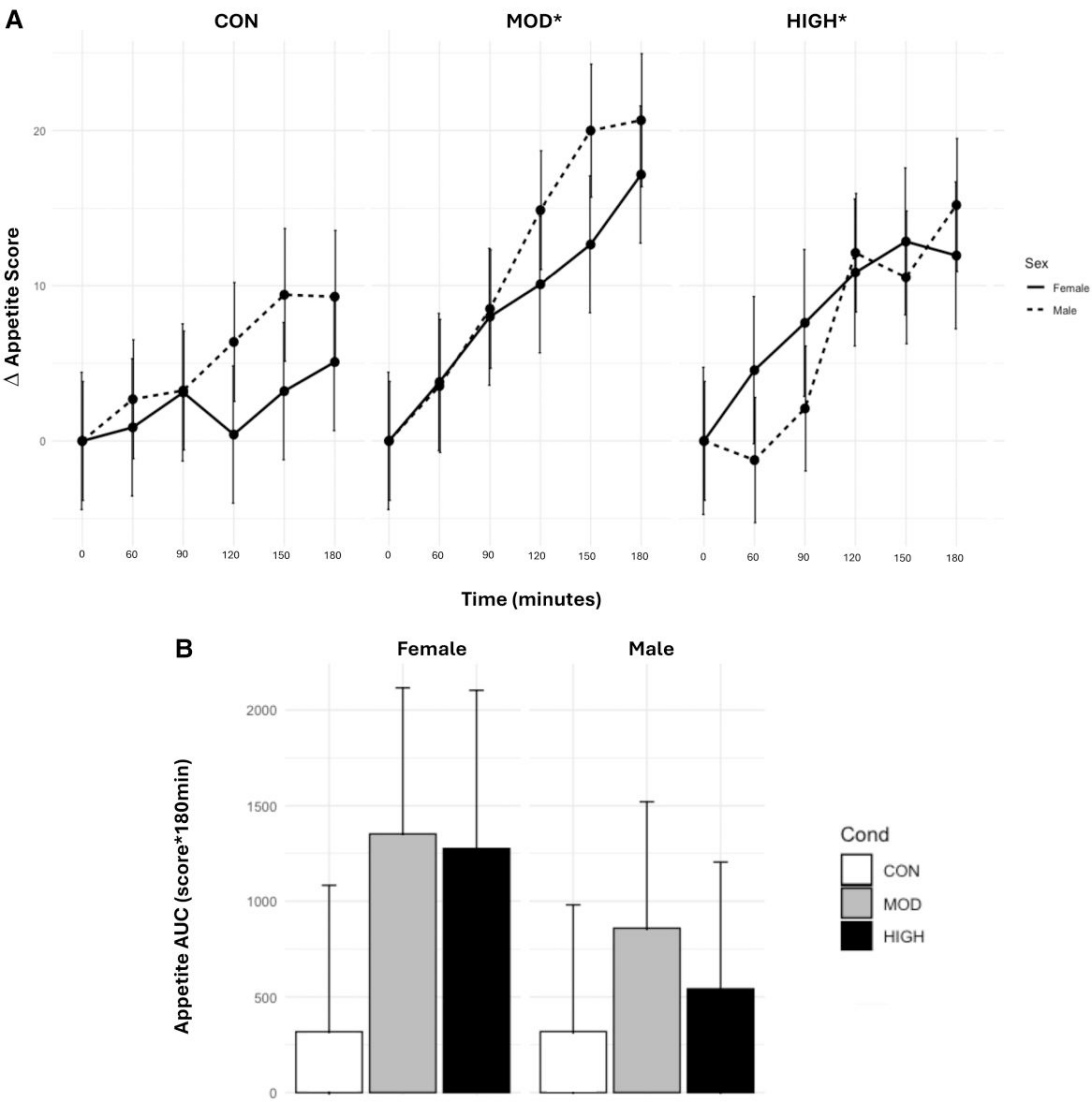
Effect of condition on (A) APP_TIMEPOINT_ and (B) APP_AUC_. Data are mean ± SE. The statistical analysis revealed there were significant main effects for condition (*P* < .001) and time (*P* < .0001). Appetite scores were significantly lower in CON than in MOD and HIGH. APP scores were significantly lower at 120, 150, and 180 minutes compared with baseline (*P* < .0001). *Significantly different from CON, *P* < .05.

There were significant main effects for condition (*P* < .001) and time (*P* < .0001). Appetite scores were significantly lower in CON (Δ3.64 ± 1.90) than in MOD (Δ9.94 ± 1.91, *P* = .001) and HIGH (Δ7.28 ± 1.95, *P* = .04). APP scores were significantly lower at 120 (*P* = .0003), 150 (*P* < .0001), and 180 minutes (*P* < .0001) compared with baseline. There were no significant main or interaction effects for APP_AUC_ (*P* = .37-.82) (Fig. 7).

### Correlations

Correlations are listed in Table 2. For the total sample, all isoforms of ghrelin were positively correlated to HUN and EAT (*P* < .0001-.01). AG and TG were negatively correlated with SAT and FULL (*P* < .0001-.03), and positively related to APP (.007-.05). Lactate was inversely related to TG (*P* = .04) and with AG (*P* = .07).

**Table 2.** Correlations between variables of the total, male, and female sample

Total	LAC	HUN	SAT	FULL	EAT	APP	BF%
TG	−.34^a^	.46^b^	−.33^a^	−.41^b^	.47^b^	.31^a^	.01
AG	−.31	.58^c^	.52^c^	.59^c^	.50^c^	.41^b^	−.11
DAG	−.28	.32^a^	−.17	−.24	.40^b^	.20	.12
LAC	—	−.37^a^	.11	.20	−.16	−.18	.04
BF%	.04	−.34^a^	.14	.11	−.31^a^	−.42^b^	—
**Males**							
TG	−.28	.60^b^	−.50^a^	−.55^b^	.75^c^	.70^c^	.73^c^
AG	−.32	.68^c^	−.50^a^	−.58^b^	.69^c^	.62^b^	.83^c^
DAG	−.21	.41^a^	−.41^a^	−.43^a^	.69^c^	.65^c^	−.53^b^
LAC	—	−.43	.27	.41	−.31	−.39	.23
BF%	.23	−.65^b^	.65^c^	.71^c^	−.57^b^	.67^c^	—
**Females**							
TG	−.34	.46	−.23	−.30	.45	.16	.20
AG	−.26	.52^a^	−.53^a^	−.58^a^	.41	.28	.01
DAG	−.30	.39	−.05	−.12	.43	.09	.18
LAC	—	−.35	−.05	−.02	.13	−.55^a^	.16
BF%	.16	−.54^a^	.25	.13	−.59^a^	−.65^b^	—

^a^ *P* < .05.

^b^ *P* < .01.

^c^ *P* < .001.

For male data, significant positive correlations were seen between TG and HUN (*P* = .01), EAT (*P* < .001), APP (*P* < .001), and BF% (*P* < .001), and negative correlations with SAT (*P* < .05) and FULL (*P* < .01). AG was positively correlated with HUN (*P* < .001), SAT (*P* = .003), EAT (*P* = .002), APP (*P* = .001), and BF% (*P* < .001), and negatively correlated with SAT (*P* < .05) and FULL (*P* < .01). DAG was positively correlated with HUN (*P* < .05), EAT (*P* < .001), APP (*P* < .001), and BF% (*P* < .001), and negatively correlated with SAT and FULL (both; *P* < .05).

For female data, significant positive correlations were seen between AG and HUN, and negative correlations between SAT and FULL (all *P* < .05). BF% was negatively correlated with HUN (*P* < .05), EAT (*P* < .05), and APP (*P* < .01).

## Discussion

The major findings of the present study were that exercise intensity and sex modulate plasma ghrelin levels, and exercise intensity affects perception of hunger regardless of sex. Our data demonstrated that high-intensity exercise suppressed plasma TG, AG, and DAG levels, confirming our hypothesis. This finding is reinforced by numerous other studies, as evidenced by our meta-analysis [22]. However, most previous research applied moderate-intensity exercise stimulus and measured only AG [22]. Interestingly, we found that moderate intensity either did not change ghrelin levels or led to a net increase. As our protocol utilized blood lactate to determine exercise intensities, these findings suggest that exercise above the lactate threshold may be necessary to elicit a suppression in ghrelin. This is supported by prior work which has shown highly enriched lactate receptors within the gastric fundus [23] and the ability of these receptors to block ghrelin secretion from the cells within the stomach via g-coupled receptor GPR81 [24]. It is important to note that although both TG and AG have been shown to be reduced following lactate-mediated GPR81 signaling [24], which may suggest a reduction in acylation, more work is needed to explore the activity of GOAT within this mechanism. However, it does not appear that lactate is the primary driver of suppressed ghrelin, as we report only moderate or nonsignificant correlations between lactate and all ghrelin isoforms (Table 2).

Exercise also altered the subjective perception of hunger in our cohort. With timepoint data, moderate intensity led to higher hunger scores compared with no exercise, with no differences found in the high intensity compared with the control condition. Males exhibited increased hunger scores during MOD compared with HIGH, while females had higher scores during MOD compared with CON. These findings suggest that high-intensity exercise may attenuate increases in hunger compared with a lower exercise intensity. Ghrelin may at least partially mediate this response, as we found that high-intensity exercise decreased each isoform while moderate-intensity exercise had a lesser effect, with a net increase seen in DAG levels. On the other hand, appetite remained unchanged in both exercise intensities. Additionally, we found that each ghrelin isoform in the control condition decreased over time, while hunger increased. While we did not provide a meal, there are data showing ghrelin levels are entrained to follow the mealtime rhythm, decreasing after breakfast time regardless if food is consumed [25]. The influence of other gut hormones such as polypeptide YY, glucagon-like peptide-1, and pancreatic polypeptide in response to exercise also cannot be ruled out, as they have also been shown to impact perception of hunger [26]. With the AUC data, we did not find any significant differences within any condition regarding VAS domains. The disparity between our timepoint data and AUC data may be due to how each is calculated; AUC is the total quantity over a given time using integrals, and any transient changes may be diluted in the calculation.

Ghrelin has been found to exhibit a diurnal variation, peaking before mealtimes and falling after eating [27]. Additionally, hunger and ghrelin were first linked when 1 of the fundamental studies that investigated a supraphysiological TG infusion in humans reported hunger as a side effect, and many studies replicated this result [27-29]. In addition, although data are limited, 2 studies have shown that near physiological doses did not change ad libitum food intake in lean individuals, but did increase food intake in individuals with obesity [5, 30]. Together, these data support the relationship between ghrelin and hunger. We report significant, moderate correlations between each ghrelin isoform and hunger. The relationship between appetite and ghrelin levels in our study were in the same direction as hunger, but weaker and only significantly related to AG and TG. This suggests ghrelin isoforms may be differentially involved in hunger and satiety signaling. GHSR1a receptors, which are utilized by AG, are found in the hypothalamus, yet there is no known evidence of DAG receptors. Therefore, AG may be the only isoform that interacts with the appetite center in the brain. However, limited evidence suggests that DAG may influence hunger by blocking AG-induced hypothalamic neuronal activity involved in appetite and food intake [31, 32]. When looking at each relationship grouped by sex, correlations between measures of appetite and ghrelin levels were stronger in males than females. However, we did not find any sex differences in baseline VAS scores across any domain, which is in agreement with other literature suggesting that there are no differences in the perception of appetite/hunger between males and females. Sex differences in receptor density and/or sensitivity to ghrelin and how this may affect appetite in humans are unknown. There are likely multiple mechanisms that regulate appetite and ghrelin levels that are not captured in the present study.

We also report that females had significantly higher levels of TG and DAG at baseline, and that there was a difference in the ghrelin response to exercise between males and females. In response to high-intensity exercise, both males and females exhibited reduced DAG levels, whereas only females had a significant reduction in AG. Collectively, this may suggest that there are sex differences in ghrelin release and/or degradation. A sexual dimorphism has been previously reported regarding ghrelin levels, with females having higher DAG and AG levels than males, regardless of obesity status [15, 16]. We found that females had higher baseline levels of TG and DAG than their male counterparts, with BMI and age being similar between groups. Studies have shown that estrogen can alter circulating ghrelin levels; however, results are conflicting. Estrogen has been shown to either upregulate ghrelin levels, decrease TG levels in postmenopausal women undergoing estrogen replacement therapy, or decrease AG following ovariectomy in rats [18]. Moreover, the literature suggests females have larger changes to energy-related hormones in response to exercise than men, although data specifically on ghrelin are scarce and mostly based on measures of AG alone [16]. Lastly, the role of the difference in BF% cannot be ruled out, as DAG and TG plasma levels have been found to be reduced in individuals with obesity [17, 33]. We report that females had higher levels of BF%, and only males had significant correlations between ghrelin levels and BF%. This suggests a complicated interplay between body composition, sex, and ghrelin. Although the mechanism behind this sex difference is currently unclear, the present data strengthen the need for more work to examine sex differences within gut hormones.

There are limitations of this study. Our ability to assess sex differences may be underpowered, due to our sample size of 6 in the female group. As our protocol included an overnight fast for each visit, real world application is weakened as most individuals consume mixed meals before and/or after exercise. Therefore, future work should examine the impact of meal content on each ghrelin isoform in response to exercise. In addition, the differences in fitness levels and exercise caloric expenditure between males and females may have impacted our results. There are several myokines suggested to affect appetite (eg, interleukin-6, GDF15, polypeptide YY, interleukin-7, BMP7, ANFPL-4, BNDF) that were not measured in the present study [34]. It is also possible that the changes to appetite and ghrelin levels may act independently from each other. Finally, we only tested lean subjects. As the ghrelin/GOAT axis is dysregulated in obesity [35], results of the present study may not apply to individuals with overweight or obesity.

Collectively, the results of the present study strengthen data on the role of high-intensity exercise in the reduction ghrelin and hunger. All isoforms of ghrelin may be associated with perception of hunger; however, more work is needed to determine if the relationship differs by sex. Our findings also suggest lactate may be involved in exercise-induced ghrelin suppression. Future work should focus on how obesity may modulate this pathway, and whether a chronic training program at differing exercise intensities mirror these results. This will allow for the development of precision exercise prescriptions designed to aid in reducing and/or preventing obesity and its related complications.

## Data Availability

Some or all datasets generated during and/or analyzed during the current study are not publicly available but are available from the corresponding author on reasonable request.

## Clinical Trial Registration Number

NCT04581980.

## Abbreviations

AG: acylated ghrelin
AUC: area under the curve
BF%: percent body fat
BMI: body mass index
DAG: deacylated ghrelin
GHSR1a: growth hormone secretagogue receptor 1a
GOAT: ghrelin O-acyltransferase
LT: lactate threshold
TG: total ghrelin
VAS: visual analog scale
VO_2peak_: peak oxygen consumption
